# Mediating effects of hypertension in association between household wealth disparities and diabetes among women of reproductive age: analysis of eight countries in sub-Saharan Africa

**DOI:** 10.1093/inthealth/ihae013

**Published:** 2024-02-06

**Authors:** Samuel H Nyarko, Isaac Y Addo, Castro Ayebeng, Kwamena S Dickson, Evelyn Acquah

**Affiliations:** Department of Epidemiology and Biostatistics, Arnold School of Public Health, University of South Carolina, Columbia, SC, USA; Centre for Social Research in Health, University of New South Wales, Sydney, NSW, Australia; Department of Population and Health, University of Cape Coast, Cape Coast, Ghana; Department of Research and Advocacy, Challenging Heights, Winneba, Ghana; Department of Population and Health, University of Cape Coast, Cape Coast, Ghana; Centre for Health Policy and Implementation Research, Institute of Health Research, University of Health and Allied Sciences, Ho, Ghana

**Keywords:** Africa, chronic disease, mediation analysis, non-communicable disease, socio-economic factors

## Abstract

**Background:**

Diabetes prevalence appears to be increasing in low- and middle-income countries, yet little is known about how hypertension status mediates the association between household wealth and diabetes. This study examined the mediation effects of hypertension in associations between household wealth and diabetes in eight sub-Saharan African (SSA) countries.

**Methods:**

This is a cross-sectional study of 71 577 women from recent Demographic and Health Surveys for eight SSA countries. Sample-weighted logistic regression and causal mediation analyses were conducted.

**Results:**

Of the 71 577 women, 1.1% (782) reported ever being diagnosed with diabetes. Women with diabetes were more likely to have hypertension compared with those without diabetes (54.9% vs 9.9%). The odds of diabetes were significantly higher among women with hypertension (adjusted odds ratio [OR] 5.71 [95% confidence interval {CI} 4.62 to 7.05]) and women from rich households (adjusted OR 1.65 [95% CI 1.23 to 2.22]) compared with their respective counterparts. Hypertension status mediated 27.4% of the association between household wealth and diabetes status.

**Conclusions:**

Hypertension status partly contributes to the associations between household wealth disparities and diabetes status among women in the selected countries. Further research and targeted interventions are needed to explore specific mechanisms and confounding factors related to household wealth disparities, hypertension status and diabetes prevalence in this population.

## Introduction

Health disparities have long been a concern in public health, as they reflect the unequal distribution of health outcomes across different subgroups.^1,2^ Household wealth may significantly affect an individual's health status.^3,4^ These factors may play a critical role in shaping access to healthcare, health behaviours and the adoption and maintenance of a healthy lifestyle. Moreover, household wealth disparities can exacerbate the burden of chronic diseases, with diabetes being a notable example.^5–7^ Studying diabetes is crucial because a lower household wealth status not only doubles the risk of diabetes,^5,7^ but also becomes particularly significant in sub-Saharan Africa (SSA), where poverty is widespread and disproportionately affects women.

Diabetes is a chronic condition affecting >1 in 10 adults globally.^8^ Over the past decade, its prevalence has surged, particularly in low- and middle-income countries.^9^ According to the International Diabetes Federation (IDF), the global prevalence of diabetes was estimated to be 10.5% in 2021, affecting 537 million adults ages 20–79 y, with projections indicating a significant increase to 643 million (11.3%) and 783 million (12.2%) by 2030 and 2045, respectively.^8^ Regionally, variations exist, with the Western Pacific having the highest number of diabetic patients. Africa, despite currently having the lowest number, is projected to experience the highest percentage increase (134%) to 55 million by 2045 if sufficient actions are not taken to address the situation.^8^

Notably, women exhibit a higher susceptibility to the simultaneous presence of behavioural and metabolic risk factors, heightening their vulnerability to non-communicable diseases (NCDs).^10,11^ The coexistence of NCDs such as pre-existing diabetes and hypertension among women poses risks to both reproductive health and foetal well-being.^12,13^ For instance, among reproductive-age women, diabetes during pregnancy has been associated with several maternal, infant and obstetric complications, such as worsening of diabetic retinopathy and nephropathy, hypertension, pre-eclampsia and preterm birth,^14,15^ as well as miscarriage and stillbirth, birth defects and foetal macrosomia.^14^

Furthermore, hypertension has emerged as a significant risk factor for the development of diabetes.^16^ Globally, hypertension affects approximately 1.3 billion adults ages 30–79 y and is considered a leading risk factor for cardiovascular diseases (including heart attacks and strokes) and chronic kidney disease.^17^ Thus epidemiological studies have indicated that approximately 40% of patients with type 1 diabetes mellitus (T1DM) also have hypertension.^18,19^ Likewise, hypertension is reported to occur in >80% of patients with type 2 diabetes mellitus (T2DM).^20^

The specific contribution of hypertension to household wealth status disparities in diabetes prevalence remains underresearched, particularly in the context of women within their reproductive years. While previous research has explored the association between hypertension and diabetes,^21,22^ there is a need for a better understanding of how these two conditions interact within the context of household wealth status. Using the Demographic and Health Survey (DHS) data for eight sub-Saharan African (SSA) countries, we assessed the contribution of hypertension to household wealth disparities in diabetes status among women in their reproductive years through mediation analysis. We therefore hypothesise that hypertension status will mediate the association between household wealth and diabetes status in the selected eight countries in SSA. By examining the mediation effect of hypertension between household wealth and diabetes status, the study sought to identify the mechanism through which household wealth disparities contribute to diabetes status and help design effective public health policies and interventions to mitigate the burden of diabetes and reduce health inequities.

## Methods

### Data source

This was a cross-sectional study based on data obtained from the most recent standard DHS conducted between 2014 and 2021. Eight SSA countries were included in the study: Benin, Cameroon, Gambia, Kenya, Lesotho, Madagascar, Mali and Zambia. The inclusion of the DHS of these countries was based on the availability of data on diabetes and hypertension, making them suitable for this study. The surveys involved samples of women of reproductive age (15–49 y), selected using a multistage stratified cluster sampling procedure to ensure national representation. This involved randomly selecting primary sampling units—mainly clusters—in the first stage and subsequently selecting various households from each of these sampling units in the second stage. The primary sample of analysis consisted of 71 557 women between the ages of 15 and 49 y who had information on diabetes and hypertension.

### Study variables

The study outcome of interest was diabetes status. The respondents were asked whether they had ever been told by a doctor or health worker that they had elevated blood sugar or diabetes, with a yes or no response. The primary exposure variable was household wealth index status, with multiple levels: poorer, poor, middle, rich and richer. These levels were then collapsed into poor, middle and rich to maximize the number of observations in these levels for stronger statistical analysis. Respondent's hypertension status was considered as a mediator in the analysis and was obtained from the question about whether respondents had ever been told by a doctor or health worker that they had elevated blood pressure or hypertension, soliciting a yes or no response.

Multiple potential confounding factors informed by theoretical and empirical literature relating to diabetes status^23–26^ were included in the study as covariates. These included age (15–19, 20–29, 30–39, 40–49 y), educational attainment (no education, primary, secondary/higher), marital status (never in a union, married/cohabitation, widowed/divorced/separated), place of residence (rural, urban) and country of residence (Benin, Cameroon, Gambia, Kenya, Lesotho, Madagascar, Mali and Zambia).

### Statistical analysis

We conducted a descriptive analysis of the background characteristics of the respondents by diabetes status. Multivariable logistic regression models were used to separately examine the association between hypertension status (the mediator) and the outcome variable (diabetes status) as well as between household wealth status (the exposure variable) and the outcome variable. The models were used to calculate unadjusted and adjusted odds ratios (ORs) with 95% confidence intervals (CIs). Furthermore, we used these models to conduct a causal mediation analysis using the ‘mediation’ package in R programming language^27^ (R Foundation for Statistical Computing, Vienna, Austria) to examine the extent to which hypertension status mediates the association between household wealth and diabetes status. The effect of hypertension compared with non-hypertension was calculated using non-hypertension status as the reference. The parameters calculated included the average causal mediation effect (ACME), average direct effect (ADE) and the proportion mediated by hypertension status with 95% CIs. All the models were adjusted for age, education, marital status, place and country of residence. A multicollinearity test was initially conducted among the predictor variables (Supplementary Table S1). Owing to the complex sampling design of the surveys, all analyses (except the causal mediation analysis, which was sample weighted to address any oversampling and undersampling in the total sample) were adjusted for clustering at the primary sampling unit level, stratification and sample weight effects. All analyses were performed using the R statistical programming language (version 4.1.3).

## Results

### Background characteristics of respondents by diabetes status

Respondents ages 30–39 y (32.8%) had higher levels of diabetes than those ages 15–19 y (6.7%). Respondents with secondary or higher education (56.3%) had higher levels of diabetes than those with no education (15.8%). The proportion of diabetes among respondents who were married (69.2%) was higher compared with those who were divorced (14.3%). Respondents from the rich household wealth index category (67.7%) had a higher proportion of diabetes compared with those from the middle household wealth index category (14.9%). The proportion of diabetes among residents in urban areas (65.8%) was higher compared with residents of rural areas (34.2%). More than half of the respondents with diabetes did not have hypertension (54.1%) while 45.9% had hypertension. Respondents from Cameroon (25.0%) and Kenya (23.9%) had higher levels of diabetes compared with the other countries (see Table 1).

**Table 1.** Characteristics of respondents by diabetes status

Characteristics	Diabetes (n=782), n (%)	No diabetes (n=70 795), n (%)
Age (years)		
15–19	52 (6.7)	14 544 (21.0)
20–29	223 (28.8)	25 597 (37.0)
30–39	254 (32.8)	18 046 (26.1)
40–49	245 (31.7)	10 917 (15.8)
Education		
No education	123 (15.8)	12 742 (18.4)
Primary	215 (27.8)	24 993 (36.2)
Secondary/higher	436 (56.3)	31 368 (45.4)
Marital status		
Never in union	128 (16.5)	20 311 (29.4)
Married/cohabiting	535 (69.2)	41 741 (60.4)
Widowed/divorced/separated	110 (14.3)	7051 (10.2)
Household wealth		
Poor	134 (17.3)	23 774 (34.4)
Middle	115 (14.9)	13 329 (19.3)
Rich	524 (67.7)	32 000 (46.3)
Residence		
Rural	265 (34.2)	37 993 (55.0)
Urban	508 (65.8)	31 110 (45.0)
Hypertension status		
No	418 (54.1)	62 447 (90.4)
Yes	355 (45.9)	6656 (9.6)
Country		
Benin	83 (10.7)	7623 (11.0)
Cameroon	193 (25.0)	13 422 (19.4)
Gambia	81 (10.5)	6105 (8.8)
Kenya	185 (23.9)	14 434 (20.9)
Lesotho	51 (6.5)	4298 (6.2)
Madagascar	60 (7.8)	9537 (13.8)
Mali	52 (6.7)	70 (0.1)
Zambia	68 (8.8)	13 615 (19.7)

All analyses are significant at p<0.001.

Multivariate analysis was conducted to determine the association between hypertension and diabetes. The unadjusted odds of diabetes were almost 8-fold among respondents with hypertension (unadjusted OR 7.96 [95% CI 6.64 to 9.55]) compared with those without hypertension (see Table 2). Respondents who had hypertension were >5-fold more likely (adjusted OR 5.71 [95% CI 4.62 to 7.05]) to have diabetes compared with those without hypertension after adjustment for sociodemographic characteristics.

**Table 2.** Association between hypertension and diabetes status

Hypertension	Unadjusted OR (95% CI)	Adjusted OR (95% CI)
No	Ref	Ref
Yes	7.96 (6.64 to 9.55)	5.71 (4.62 to 7.05)*

Ref: reference category.

*p<0.05.

Further analysis was conducted to determine the relationship between household wealth and diabetes. The result showed that household wealth was significantly associated with diabetes, with 2.90-fold unadjusted odds of diabetes among respondents from rich households (unadjusted OR 2.90 [95% CI 2.32 to 3.63]) compared with those from poor households. The OR of diabetes was 65% higher among respondents from rich households (adjusted OR 1.65 [95% CI 1.23 to 2.22]) compared with those from poor households after adjustment for sociodemographic characteristics. Age, education, marital status, place of residence and country of residence were significantly associated with diabetes. Those ages 40–49 y (adjusted OR 5.80 [95% CI 3.62 to 9.28]) were more likely to have diabetes compared with those ages 15–19 y. The likelihood of diabetes was higher among those with higher education (adjusted OR 1.89 [95% CI 1.37 to 2.59]) compared with those with no education. Those who had divorced had a higher likelihood of diabetes (adjusted OR 1.53 [95% CI 1.01 to 2.34]) compared with those who were married. Those in urban areas (adjusted OR 1.60 [95% CI 1.25 to 2.05]) had higher odds of diabetes compared with those in rural areas. Regarding country of residence, those in Mali were more likely to have diabetes (adjusted OR 54.6 [95% CI 34.0 to 87.6]) compared with those in Benin. Those in Zambia had the lowest likelihood of diabetes compared with those in Benin (adjusted OR 0.35 [95% CI 0.23 to 0.53]) (see Table 3).

**Table 3.** Association between household wealth and diabetes status

Characteristic	Unadjusted OR (95% CI)	Adjusted OR (95% CI)
Household wealth		
Poor	Ref	Ref
Middle	1.54 (1.13 to 2.08)	1.24 (0.90 to 1.70)
Rich	2.90 (2.32 to 3.63)	1.65 (1.23 to 2.22)*
Age (years)		
15–19		Ref
20–29		2.00 (1.27 to 3.16)*
30–39		3.31 (2.05 to 5.33)*
40–49		5.80 (3.62 to 9.28)*
Education		
No education		Ref
Primary		1.31 (0.95 to 1.81)
Secondary/higher		1.89 (1.37 to 2.59)*
Marital status		
Never in union		Ref
Married/cohabiting		1.34 (0.95 to 1.91)
Widowed/divorced/separated		1.53 (1.01 to 2.34)*
Residence		
Rural		Ref
Urban		1.60 (1.25 to 2.05)*
Country of residence		
Benin		Ref
Cameroon		1.02 (0.74 to 1.41)
Gambia		0.92 (0.59 to 1.42)
Kenya		0.92 (0.64 to 1.33)
Lesotho		0.69 (0.46 to 1.04)
Madagascar		0.50 (0.34 to 0.75)*
Mali		54.6 (34.0 to 87.6)*
Zambia		0.35 (0.23 to 0.53)*

*p<0.05.

Table 4 presents the results of a causal mediation analysis between hypertension, household wealth and diabetes status. The results show that hypertension status significantly mediated the association between household wealth and diabetes status by contributing to an average of 27.4% of the total effect (proportion mediated [average] 0.274021 [95% CI 0.161818 to 0.61]) of the association (see Table 4).

**Table 4.** Causal mediation analysis between hypertension, household wealth and diabetes status

Parameter	Estimate	95% CI	p-Value
Total effect	0.004 820	0.002177 to 0.01	0.002*
ACME (average)	0.001 321	0.000990 to 0.00	0.000*
ADE (average)	0.003 499	0.000835 to 0.01	0.016*
Proportion mediated (average)	0.274 021	0.161818 to 0.61	0.002*

*p<0.05.

## Discussion

This study aimed to investigate the mediating effects of hypertension in the relationship between household wealth disparities and the diabetes status of women in eight SSA countries. To the best of our knowledge, this is the first parallel mediation conducted to disentangle the complex associations among household wealth disparities, hypertension and diabetes in community-dwelling women ages 15–49 y in SSA.

To effectively evaluate the mediation effects of hypertension in the associations between household wealth disparities and diabetes status, it is critical to first examine the associations between household wealth and diabetes status as well as between hypertension and diabetes status. In line with this assumption, we found significant patterns in the association between household wealth and diabetes status, with diabetes more prevalent among respondents from wealthier households. This finding demonstrates the role of affluence in diabetes incidence and can be attributed to several potential reasons. First, women from wealthier households often enjoy access to various amenities and modern conveniences that may promote a sedentary lifestyle. Prolonged sitting and reduced physical activity associated with such a lifestyle are recognised risk factors for diabetes.^28^ Second, higher disposable income in wealthier households can lead to greater purchasing power, enabling such households to easily afford or access processed and energy-dense foods. Diets rich in unhealthy fats, sugars and refined carbohydrates can contribute to weight gain and insulin resistance, thereby increasing the risk of diabetes.^29,30^ On the other hand, the analysis established a significant association between hypertension and diabetes, indicating that hypertension is also a risk factor for diabetes development in this population and vice versa. Evidence shows that hypertension and diabetes are two common chronic conditions that often coexist and are closely related.^31,32^ In T2DM, which is the most common form of diabetes,^33^ the body becomes resistant to the effects of insulin, a hormone that regulates blood sugar levels.^34–36^ Insulin resistance can lead to an increase in blood sugar levels, which in turn can damage blood vessels and contribute to hypertension.^34,35^ Also, hormones such as aldosterone and angiotensin II play essential roles in blood pressure regulation.^33,36,37^ Thus there may be alterations in the balance of these hormones, leading to an increase in blood pressure and subsequently diabetes.^36,37^ This finding is consistent with earlier studies that examined the association between diabetes and hypertension.^22,38,39^ It reinforces the importance of controlling hypertension to reduce the risk of diabetes and its associated complications.

Based on the primary hypothesis of this study, we employed various techniques in examining the mediational role of hypertension in associations between household wealth disparities and diabetes status. Specifically, we utilised estimates of the total effect, ACME, ADE and proportion mediated in understanding the role of hypertension as a mediator in the relationship between household wealth disparities and diabetes status. Consistent with our hypothesis, the statistically significant total effect suggests that household wealth is positively associated with diabetes status, meaning that as household wealth increases, the likelihood of having diabetes also increases. The ACME estimate suggests that hypertension acts as a partial mediator, explaining approximately 0.001321 of the total effect of household wealth on diabetes through an indirect pathway. This implies that hypertension plays a role in linking household wealth to diabetes incidence. On the other hand, the ADE estimate indicates that even after accounting for the mediating effect of hypertension, there remains a significant direct association between household wealth and diabetes. This suggests that a portion (approximately 0.003499) of the total effect is not influenced by hypertension, indicating the presence of other pathways or factors contributing to diabetes risk associated with household wealth. The proportion mediated estimate of 0.274021 provides additional insights into the proportion of the association between household wealth disparities and diabetes that can be explained by the mediating effect of hypertension. Approximately 27.4% of the association can be attributed to hypertension, underscoring its importance as a partial mediator in this relationship.

Drawing from the existing literature, potential confounding mediating factors in these findings may encompass dietary habits, physical inactivity, obesity, access to healthcare, levels of health literacy, genetic predisposition and psychosocial factors.^40–44^ Notwithstanding the key finding and primary focus of this present study, these potentially confounding variables may exhibit associations with both the independent variable (household wealth disparities) and the dependent variable (diabetes status). Consequently, they might have ‘masked’ genuine relationships between the primary variables of interest. The consideration of these factors in the future is of paramount importance, as their influence could impact the observed associations between household wealth and the risk of diabetes. Further research should consider exploring the causal relationships and pathways through which these potential confounding factors influence diabetes risk. Understanding these mechanisms can lead to more targeted interventions.

Besides conducting the mediation analysis, the study also provides insights into the interplay of hypertension, wealth disparities and diabetes across various sociodemographic factors. First, the study identified that respondents ages 30–39 y had the highest proportion of diabetes, indicating that middle-aged women are more susceptible to the disease. However, caution should be exercised when interpreting this finding due to the limitation of the data being confined to women ages 15–49 y. Therefore, this finding may not fully represent the entire population and extrapolation beyond this gender and age range should be approached with caution. Second, the association between higher education and an increased likelihood of diabetes suggests a potential link between education level and diabetes risk. This finding aligns with the results observed in a recent study encompassing 29 low- and middle-income countries.^9^ Possible reasons for this finding are that women with higher education levels may be more likely to have sedentary occupations, resulting in reduced physical activity levels, which in turn increases the risk of developing diabetes.^45,46^ Additionally, individuals with higher education may be more conscious of their risk factors and thus seek more frequent medical check-ups, potentially leading to earlier diabetes diagnosis.^47^ This finding emphasises the need for targeted educational campaigns to raise awareness about diabetes prevention and management among women with higher education.

Furthermore, the higher proportion of diabetes among married respondents indicates the influence of changes in lifestyles on diabetes risk. For example, new responsibilities and stressors that come with marriage can affect mental health and stress, and psychological factors are known to influence diabetes risk through hormonal and physiological pathways.^48,49^ Moreover, marriage can result in joint finances and elevated socio-economic status for women. While this can provide access to more resources, it may also lead to the consumption of higher-calorie diets, which could contribute to diabetes risk. Additionally, married women may find themselves with less time for regular physical activity due to family responsibilities, leading to a more sedentary lifestyle. These combined factors can contribute to a higher prevalence of diabetes among married women compared with their unmarried counterparts. This result aligns with a previous study that indicated being widowed was associated with a decreased risk of T2DM when compared with being married.^50^ This suggests the importance of incorporating family and marital dynamics into diabetes prevention and management programs.

Also, our findings indicated a higher prevalence of diabetes among participants from urban areas, suggesting the potential roles of urbanisation and affluence in diabetes incidence. This finding supports a systematic review and meta-analysis conducted by Uthman et al.,^51^ which focused on the global prevalence and trends of hypertension and T2DM among slum residents. It is well established that urban areas and higher socio-economic status often come with increased job demands and stress levels. Chronic stress and its impact on mental health can lead to hormonal imbalances, potentially influencing the risk of diabetes, as suggested by previous studies.^52–54^

### Implications for policy and research

The study's findings emphasise the importance of targeted public health interventions to address hypertension and diabetes in SSA. Developing and implementing targeted intervention programs that address both hypertension and diabetes within the context of household wealth disparities is essential. By addressing hypertension as a mediating factor, policies can aim to break the link between socio-economic inequalities and diabetes, promoting a holistic approach to women's health.

Promoting integrated healthcare services that consider both hypertension and diabetes management within the same framework can enhance patient outcomes. This approach can be implemented at various healthcare levels, emphasizing a coordinated effort among primary care providers, specialists and community health workers.

The association between household wealth and diabetes highlights the necessity to address household wealth disparities. Economic policies aimed at reducing wealth disparities and improving access to healthcare facilities may contribute to a decrease in diabetes incidence. Additionally, since hypertension appears to partially mediate the relationship between household wealth and diabetes, policies prioritising hypertension management and control could positively impact diabetes incidence in SSA.

While this research provides valuable insights, future studies should explore additional factors that may mediate the association between household wealth disparities and diabetes. Understanding other potential mediators can lead to more targeted interventions and better policy recommendations. Policymakers and researchers should also consider the specific contexts and nuances of different African countries when formulating diabetes prevention and management strategies. The findings may vary across regions with varying levels of economic development and healthcare infrastructure. Taking these factors into account will lead to more effective and context-specific policies aimed at reducing diabetes incidence in SSA.

### Strengths and limitations

The study has several strengths. First, the study utilised data from the most recent standard DHS conducted in eight SSA countries. These surveys are known for providing reliable data on NCDs, including diabetes and hypertension, resulting in a comprehensive and relevant dataset for the study. Additionally, the primary sample of analysis consisted of 71 557 women ages 15–49 y, ensuring a substantial sample size that enhances the statistical power and robustness of the findings. Furthermore, the study employed multivariate logistic regression models to examine the association between hypertension status and diabetes, as well as between household wealth status and diabetes. This analytical approach enabled the control of potential confounding factors, leading to more accurate and reliable results. Additionally, the study conducted a causal mediation analysis using the ‘mediation’ package in R programming language. This methodology allowed for investigating the mediating role of hypertension status in the association between household wealth and diabetes, providing valuable insights into the underlying mechanisms. The analyses also adjusted for the complex sampling design of the surveys, considering clustering at the primary sampling unit level, stratification and sample weight effects. This adjustment helped to mitigate potential biases in the estimates and ensured the validity of the conclusions.

Nevertheless, there are some limitations to consider. First, the study's reliance on cross-sectional data limits its ability to establish causal relationships between variables. Longitudinal studies would be more suitable for exploring causality over time. Additionally, the study relied on self-reported data for diabetes and hypertension status, which may introduce recall bias or underreporting. Thus future studies could benefit from objective measures such as medical records or clinical assessments. Also, the current study was restricted to respondents ages 15–49 y. However, diabetes is more prevalent among older individuals and therefore the findings cannot be generalised. Moreover, while the study focused on eight SSA countries, caution should be exercised when generalising the findings to the entire continent or other global regions, as they may not be fully representative. Despite controlling for various covariates, unmeasured or residual confounding factors may still influence the observed associations. Furthermore, using data from surveys conducted between 2014 and 2021 may not capture recent changes in diabetes and hypertension prevalence or other relevant factors that could influence the results. Finally, causal mediation analysis relies on certain assumptions, such as no unmeasured confounding and no effect modification, which should be carefully considered when interpreting the mediation results.

## Conclusions

The causal mediation analysis presented in this study underscores the significant role of hypertension as a mediator in the link between household wealth disparities and diabetes incidence. Of the association, 27.4% can be attributed to hypertension, underscoring its importance as a partial mediator in this relationship. These findings hold valuable implications for policymakers, as they can use this understanding to develop targeted interventions aimed at addressing hypertension and reducing diabetes risk, particularly among populations with varying household wealth levels. However, to further enhance our understanding, future research should investigate other potential mediators and consider the broader social determinants of health in diabetes prevention efforts. Embracing a comprehensive and equitable approach to healthcare will be vital in effectively addressing diabetes in SSA.

## Supplementary Material

ihae013_Supplemental_File

## Data Availability

Data for the current study are accessible at the DHS data repository (https://dhsprogram.com/data/available-datasets.cfm).
