# Supplementary material for: Mediating effects of hypertension in association between household wealth disparities and diabetes among women of reproductive age: analysis of eight countries in sub-Saharan Africa
Source: Int Health. 2024 Feb 6;17(1):41–8. doi: 10.1093/inthealth/ihae013 (PMC11697155; doi:10.1093/inthealth/ihae013)
Supplement: ihae013_Supplemental_File [file ihae013_supplemental_file.docx]

**Supplementary Table 1. Multicollinearity test among study variables**

| Parameter | GVIF | Df | GVIF^(1/(2*Df)) |
| --- | --- | --- | --- |
| Household wealth | 1.695840 | 2 | 1.141159 |
| Hypertension status | 1.083603 | 1 | 1.040962 |
| Age | 1.572940 | 3 | 1.078414 |
| Education | 1.679040 | 2 | 1.138322 |
| Marital status | 1.581362 | 2 | 1.121393 |
| Residence | 1.584771 | 1 | 1.258877 |
| Country | 1.522149 | 7 | 1.030464 |

GVIF, Generalized Variance Inflation Factor.
